# Diverse Stomatal Behaviors Mediating Photosynthetic Acclimation to Low Temperatures in *Hordeum vulgare*

**DOI:** 10.3389/fpls.2018.01963

**Published:** 2019-01-09

**Authors:** Barbara Jurczyk, Maciej Grzesiak, Ewa Pociecha, Magdalena Wlazło, Marcin Rapacz

**Affiliations:** ^1^Department of Plant Physiology, Faculty of Agriculture and Economics, University of Agriculture in Kraków, Kraków, Poland; ^2^Institute of Plant Physiology, Polish Academy of Sciences, Kraków, Poland

**Keywords:** chlorophyll fluorescence, cold acclimation, prehardening, Rubisco activase, stomatal conductance

## Abstract

Photosynthetic acclimation to cold conditions is an important factor influencing freezing tolerance of plants. Photosynthetic enzyme activities increase as part of a photochemical mechanism underlying photosynthetic acclimation to low temperatures. Additionally, a non-photochemical mechanism may be activated to minimize photooxidative damage. The aim of this study was to test the hypothesis that differences in stomatal conductance in *Hordeum vulgare* plants with contrasting freezing tolerances induce various strategies for photosynthetic acclimation to cold stress. Different stomatal behaviors during the prehardening step resulted in diverse plant reactions to low-temperature stress. Plants with a relatively low freezing tolerance exhibited decreased stomatal conductance, resulting in decreased photochemical activity, faster induction of the non-photochemical mechanism, and downregulated expression of two Rubisco activase (*Rca*A) splicing variants. In contrast, plants with a relatively high freezing tolerance that underwent a prehardening step maintained the stomatal conductance at control level and exhibited delayed photochemical activity and *Rca*A expression decrease, and increased Rubisco activity, which increased net photosynthetic rate. Thus, in barley, the induction of photoinhibition avoidance (i.e., non-photochemical photoacclimation mechanism) is insufficient for an effective cold acclimation. An increase in cold-induced net photosynthetic rate due to open stomata is also necessary.

## Introduction

Optimal photosynthetic performance requires a balance between energy input and energy consumption. When plants are exposed to low temperatures, the balance between “source” and “sink” processes (photostasis) may be disturbed, ultimately resulting in photoinhibition. To protect photosynthetic apparatus from photoinhibition induced by low-temperature stress, plants have evolved protective mechanisms that help maintain cellular photostasis (Ensminger et al., 2006). Photochemical and non-photochemical mechanisms underlying photosynthetic acclimation to cold stress have been identified. The photochemical mechanism depends on the enhancement of photosynthetic metabolism, while the non-photochemical mechanism relies on the dissipation of excess energy. Both mechanisms may be activated depending on the freezing tolerance level (Hurry et al., 1995; Jurczyk et al., 2016a).

The catalytic activity of the main photosynthetic enzyme [ribulose-1,5-bisphosphate carboxylase/oxygenase (Rubisco)] is induced and maintained by Rubisco activase. In barley, two Rubisco activase isoforms (42 and 46 kDa) are encoded by two tandemly oriented genes, *Rca*A and *Rca*B. The differential splicing of *Rca*A generates two Rubisco activase isoforms, while *Rca*B encodes only the smaller peptide (Rundle and Zielinski, 1991).

During cold acclimation, photosynthetic enzyme activities reportedly increase (Hurry et al., 1995) as a part of the aforementioned photochemical mechanism typical of herbaceous winter plants. RA in response to low-temperature stress was observed to increase more in freezing-tolerant *Lolium perenne* plants than in plants exhibiting lower freezing tolerance (Jurczyk et al., 2016a). The induced production of the large Rubisco activase isoform in the freezing-tolerant *L. perenne* genotype is important for the photochemical mechanism responsible for photosynthetic acclimation to cold stress (Jurczyk et al., 2016a).

At low temperatures, plants dehydrate because of restricted water uptake. Under these conditions, rapid stomatal closure (i.e., hours after first being exposed to cold stress) limits water loss. This mechanism occurs in cold-tolerant, but not cold-sensitive species (Wilkinson et al., 2001). Additionally, after a prolonged exposure to cold (i.e., days), the net assimilation rate and CO_2_ exchange rate are reportedly higher in winter (more hardy) cultivars than in spring cultivars (Hurry et al., 1995). This contrasting stomatal behavior reflects the reaction of plants to low-temperature stress rather than acclimation process. Furthermore, these results suggest that the regulation of the stomatal aperture is important for the tolerance of plants to low temperatures.

The objective of this study was to test the hypothesis that differences in stomatal conductance in *Hordeum vulgare* plants with contrasting freezing tolerance induce diverse strategies for photosynthetic acclimation to cold conditions. The following aspects of photosynthetic acclimation were investigated: activation of photochemical and non-photochemical mechanisms; net photosynthetic rate and stomatal conductance; photochemical activity of photosystem II (PSII); RA; and the expression of both transcript variants of alternatively spliced barley *Rca*A gene.

## Materials and Methods

### Plant Materials and Stress Treatments

This study involved the following two barley lines: POA_7333/06-1 and POA_7183/06-6. Line POA_7333/06-1 (MFT) is more freezing-tolerant than line POA_7183/06-6 (LFT). Mean survival after freezing assessed in two field-laboratory and one laboratory experiments were 66.5 vs. 32.3%, respectively (Fiust and Rapacz, unpublished). Seeds from the Strzelce Plant Breeding collection were germinated at 28°C in darkness. After 3 days, the germinated seeds of each line were transferred to 12 pots (20-cm diameter) containing equal volumes of sand, soil, and peat. Plants were grown under controlled-environment conditions [18°C, 14-h light/10-h dark photoperiod, 250 μmol m^-2^ s^-1^ photosynthetically active radiation (PAR) provided by Agro HPS lamps (Philips, Brussels, Belgium)]. After 4 weeks, plants were divided into two groups as follows. The plants in six pots were maintained at control conditions (18°C), while plants in the remaining six pots were prehardened for 2 weeks (15/12°C, 14-h light/10-h dark photoperiod, 250 μmol m^-2^ s^-1^ PAR) before being cold acclimated for 3 weeks (4/2°C, 14-h light/10-h photoperiod, 250 μmol m^-2^ s^-1^ PAR). Plants were analyzed before the low-temperature treatment (C0), after prehardening (PH/2), and after 1 and 3 weeks of cold acclimation (CA1/3 and CA3/5). Control plants were analyzed at the same time points.

### Rubisco Activity

Ribulose-1,5-bisphosphate carboxylase/oxygenase (EC 4.1.1.39) activity was assayed as described by Sharkey et al. (1991). In this method, enzyme activity is coupled to NADH oxidation *via* 3-phosphoglycerate kinase and glyceraldehyde 3-phosphate dehydrogenase. The oxidation of NADH is continuously monitored with a spectrophotometer. Samples were collected from the middle part of leaves and their area was measured using the Cl-202 Leaf Area Meter (CID Bio-Science, United States). Samples were immediately frozen in liquid nitrogen and analyzed with the Ultrospec 2100 Pro spectrophotometer (Biosciences Amersham, Sweden) as described by Jurczyk et al. (2016b). Data are presented as the mean of six independent measurements.

### Net Photosynthetic Rate and Stomatal Conductance

Rate of leaf gas exchange parameters (P_n_ and G_s_) was measured using CO_2_ infrared (IRGA) gas analyzer (CIRAS-2, PP System, Amesbury, United States) with Parkinson’s assimilation chamber (narrow-leaf type) and with light attachment PLC6(U) – Automatic Universal Light Unit. During the measurements an open system was used. A flow rate of ambient air with a constant CO_2_ concentration (380 μmol mol^-1^) through the assimilation chamber amounted to 0.5 dm^3^ min^-1^. The chamber temperature was kept below 25°C until the photosynthesis rate was stabilized. Photosynthetic capacity at light saturation was reached by exposing leaves to PAR at 1000 μmol m^-2^ s^-1^. The measurements were taken in the central part of mature leaves.

Data are presented as the mean of six independent measurements.

### Chlorophyll Fluorescence Parameters

Chlorophyll *a* fluorescence signals in plants were measured in a growth chamber with a modulated FMS2 fluorescence monitoring system (Hansatech, King’s Lynn, United Kingdom). The central fragments of the second leaf were adapted to darkness for 30 min using leaf clips before being analyzed as described by Janeczko et al. (2016). A saturating light (6,500 μmol m^-2^ s^-1^ PAR) was applied along with actinic light (500 μmol m^-2^ s^-1^ PAR). The fluorescence signal was stabilized under actinic light for 5 min. The following parameters were analyzed: F_v_/F_m_ (maximum quantum yield of PSII), Φ_PSII_ (quantum yield of the electron transport in PSII), q_P_ (photochemical quenching coefficient), and NPQ (non-photochemical quenching coefficient) (Genty et al., 1989; Bilger and Björkman, 1991). Data are presented as the mean of 10 independent measurements.

### Analysis of *Rca*A1 and *Rca*A2 Transcript Accumulation

The transcript levels of two splicing variants of *Rca*A, *Rca*A1, and *Rca*A2 (Rundle and Zielinski, 1991), were determined. A quantitative reverse transcription polymerase chain reaction (qRT-PCR) analysis was completed using the 7500 real-time PCR system (Applied Biosystems, Foster City, CA, United States). Samples (approximately 0.05 g central part of the second leaf) were collected and frozen in liquid nitrogen, after which mRNA was extracted with the RNeasy Plant Mini Kit (Qiagen, Hilden, Germany). Approximately 500 ng RNA template was used for each reverse transcription reaction with the QuantiTect Reverse Transcription Kit (Qiagen). The gDNA Wipeout Buffer (included in Reverse Transcription Kit) was used to eliminate any contaminating genomic DNA. The concentration and quality of the RNA and cDNA were determined with the Q5000 UV-Vis spectrophotometer (Quawell, San Jose, CA, United States). The subsequent qRT-PCR was completed as described by Jurczyk et al. (2012). Primers and probes were designed using the Primer Express program (version 3.0.1) (Applied Biosystems, Foster City, CA, United States) to specifically amplify *Rca*A1, *Rca*A2, and an endogenous reference gene, *Actin* (An et al., 1996). The primers and probe for *Rca*A2 targeted the remaining part of intron 5, while the probe for *Rca*A1 targeted the exon–exon junction (Rundle and Zielinski, 1991). Primer and probe sequences as well as GenBank sequences are presented in Table 1. The qRT-PCR data were analyzed using the 7500 real-time PCR Sequence Detection Software (version 1.3) (Applied Biosystems). The *Rca*A1 and *Rca*A2 expression levels were determined relative to that of *Actin*. Data are presented as the mean of five biological replicates, with four technical replicates each.

**Table 1:** Sequence origins and primers and probes sequences used in the study.

Gene name	GenBank ID	Forward primer	Reverse primer	TaqMan MGB probe
*Rca*A1	M55446.1	CTAACCAGGATGCGATGAAGACT	GGCACGGGCAAAGTACCTT	FAM-TTCTACGGTAAAGGAGCACAG-MGB
*Rca*A2	M55447.1	TCCATACAACACCCACCATCTC	CAAAGTACCTTGCTGTGCTCCTT	FAM-TGCTGCATAGGAGGAG-MGB
*Actin*	AY145451.1	CAATGTTCCTGCCATGTACG	AGCGAGATCCAAACGAAGAA	FAM- CCCTCTATGCAAGTGGTCGT-MGB


### Statistical Analysis

The general effects of prehardening and cold acclimation were tested using a two-way ANOVA (temperature treatment and barley line as factors) at *P* = 0.05. The effect of temperature on a particular barley line at each time point was tested using a one-way ANOVA (temperature as a factor) at *P* = 0.05. Temporal changes for a particular barley line were calculated using Duncan’s multiple range test. All data analyses were completed using STATISTICA (version 10.0) (Stat Soft, Inc., Tulsa, OK, United States 2011). Principal component analysis (PCA) based on eigenvalue decomposition of data correlation matrix was performed to present relationships in the different physiological characteristics after cold response.

## Results

### Net Photosynthetic Rate and Stomatal Conductance

**FIGURE 1:**
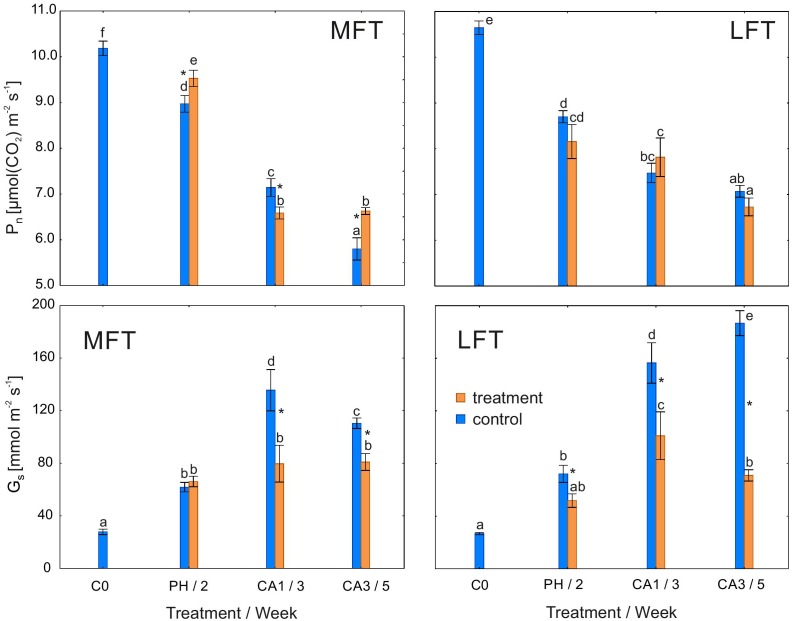
Changes in potential capacities of net photosynthetic rate (P_n_) and stomatal conductance (G_s_) of *Hordeum* *vulgare* plants after the prehardening and cold acclimation steps. Data are presented as the mean ± standard error. Asterisks indicate values that are different between the treated and control plants according to one-way ANOVA (*P* = 0.05). Values marked with the same letter are not significantly different (*P* = 0.05) according to Duncan’s multiple range test. LFT and MFT, low and high freezing-tolerant plants, respectively. Measurements were taken before the prehardening step (C0; 18°C, 14-h light/10-h dark photoperiod, 250 μmol m^-2^ s^-1^ PAR), after 2 weeks of prehardening (PH/2; 15/12°C, 14-h light/10-h dark photoperiod, 250 μmol m^-2^ s^-1^ PAR), and after 1 (CA1/3) and 3 (CA3/5) weeks of cold acclimation (4/2°C, 14-h light/10-h dark photoperiod, 250 μmol m^-2^ s^-1^ PAR).

P_n_ was higher in MFT plants than in the controls after the prehardening step and after 3 weeks of cold acclimation (Figure 1). However, after 1 week of cold acclimation, P_n_ was lower in MFT plants than in the controls. In contrast, there were no differences in P_n_ of the cold-treated and control LFT plants. In all plants, P_n_ after the cold acclimation was lower than before the prehardening step (C0). Meanwhile, G_s_ was lower in the cold-acclimated plants than in the controls for both barley lines. G_s_ decreased after the prehardening step relative to the control G_s_ only in the LFT line (Figure 1). In all plants, G_s_ was higher after the cold acclimation than at C0. Infrared gas analyser data was presented in Supplementary Table S1.

### Photochemical Activity of PSII

**FIGURE 2:**
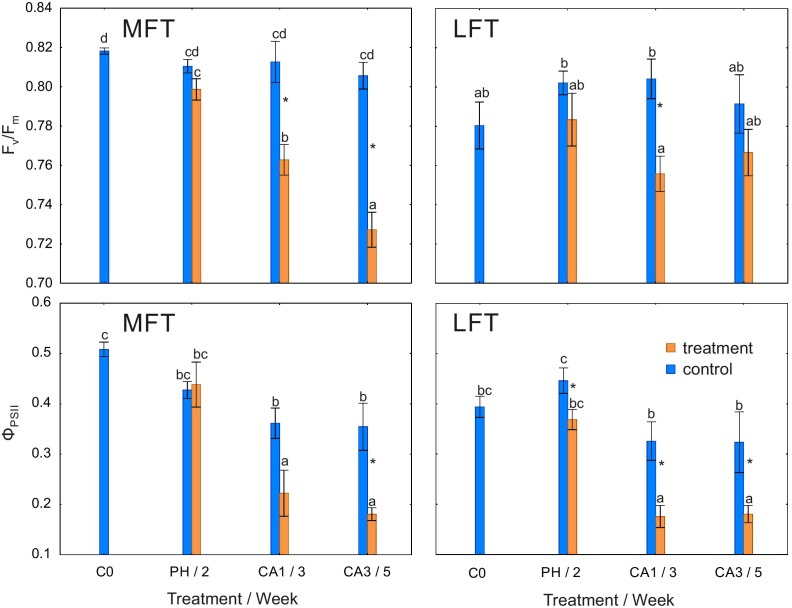
Changes in the maximum quantum yield of PSII (F_v_/F_m_) and the quantum yield of electron transport in PSII (Φ_PSII_) of *Hordeum* *vulgare* plants after the prehardening and cold acclimation steps. Data are presented as the mean ± standard error. Asterisks indicate values that are different between the treated and control plants according to a one-way ANOVA (*P* = 0.05). Values marked with the same letter are not significantly different (*P* = 0.05) according to Duncan’s multiple range test. LFT and MFT, low and high freezing-tolerant plants, respectively. Measurements were taken before the prehardening step (C0; 18°C, 14-h light/10-h dark photoperiod, 250 μmol m^-2^ s^-1^ PAR), after 2 weeks of prehardening (PH/2; 15/12°C, 14-h light/10-h dark photoperiod, 250 μmol m^-2^ s^-1^ PAR), and after 1 (CA1/3) and 3 (CA3/5) weeks of cold acclimation (4/2°C, 14-h light/10-h dark photoperiod, 250 μmol m^-2^ s^-1^ PAR).

F_v_/F_m_ was lower in the cold-acclimated MFT plants than in the non-cold-acclimated plants after 1 and 3 weeks (Figure 2). A decrease in F_v_/F_m_ of treated LFT plants was detected only after 1 week of cold acclimation. F_v_/F_m_ of the control plants of both lines as well as the treated LFT plants was unchanged during the experiment, while that of the treated MFT plants progressively decreased during the experiment (Figure 2).

Φ_PSII_ decreased in treated LFT plants during the whole experiment (Figure 2). This value decreased in the treated MFT plants only after 3 weeks of cold acclimation. In both treated lines as well as in control LFT plants, Φ_PSII_ decreased between the prehardening step and 1 week after initiating the cold acclimation (Figure 2).

**FIGURE 3:**
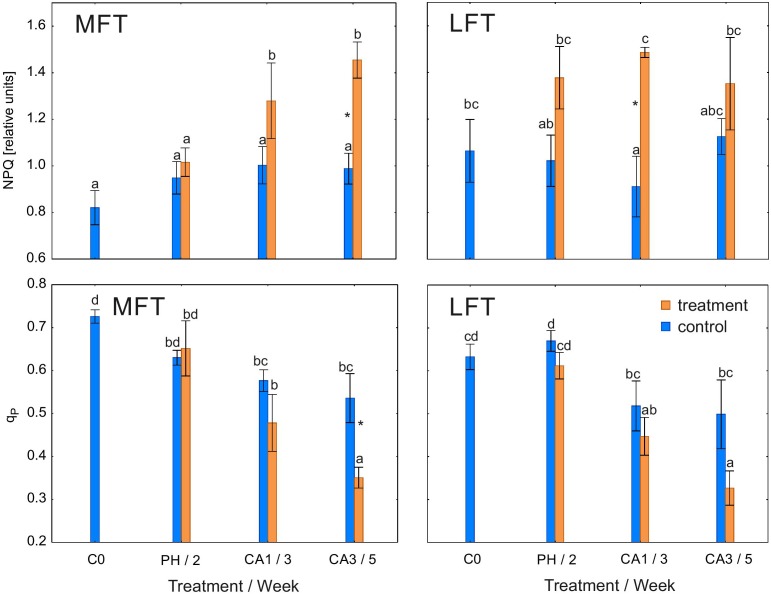
Changes in the non-photochemical quenching coefficient (NPQ) and photochemical quenching coefficient (q_P_) of *Hordeum* *vulgare* plants after the prehardening and cold acclimation steps. Data are presented as the mean ± standard error. Asterisks indicate values that are different between the treated and control plants according to a one-way ANOVA (*P* = 0.05). Values marked with the same letter are not significantly different (*P* = 0.05) according to Duncan’s multiple range test. LFT and MFT, low and high freezing-tolerant plants, respectively. Measurements were taken before the prehardening step (C0; 18°C, 14-h light/10-h dark photoperiod, 250 μmol m^-2^ s^-1^ PAR), after 2 weeks of prehardening (PH/2, 15/12°C, 14-h light/10-h dark photoperiod, 250 μmol m^-2^ s^-1^ PAR), and after one (CA1/3) and three (CA3/5) weeks of cold acclimation (4/2°C, 14-h light/10-h dark photoperiod, 250 μmol m^-2^ s^-1^ PAR).

Non-photochemical quenching coefficient was higher in treated LFT and MFT plants than in controls after 1 or 3 weeks of cold acclimation (Figure 3). Additionally, NPQ was stable during the experiment in control plants and treated LFT plants. In the MFT plants, NPQ increased between the prehardening step and 1 week after initiating the cold acclimation.

Q_p_ was lower in MFT plants than in the controls after 3 weeks of cold acclimation (Figure 3). However, q_p_ was unaffected by the treatment in LFT plants. During the experiment, q_p_ decreased in both treated lines as well as in the control MFT lines. In the control LFT plants, q_p_ at the end of the experiment was similar to that before the experiment.

### Rubisco Activity and *Rca*A1 and *Rca*A2 Expression

**FIGURE 4:**
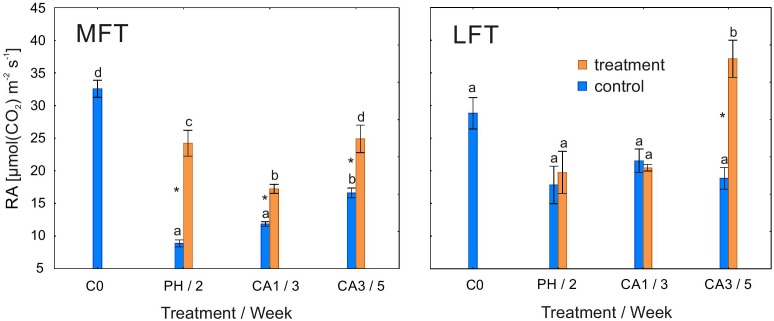
Changes in the RA of *Hordeum* *vulgare* plants after the prehardening and cold acclimation steps. Data are presented as the mean ± standard error. Asterisks indicate values that are different between the treated and control plants according to a one-way ANOVA (*P* = 0.05). Values marked with the same letter are not significantly different (*P* = 0.05) according to Duncan’s multiple range test. LFT and MFT, low and high freezing-tolerant plants, respectively. Measurements were taken before the prehardening step (C0; 18°C, 14-h light/10-h dark photoperiod, 250 μmol m^-2^ s^-1^ PAR), after 2 weeks of prehardening (PH/2; 15/12°C, 14-h light/10-h dark photoperiod, 250 μmol m^-2^ s^-1^ PAR), and after 1 (CA1/3) and 3 (CA3/5) weeks of cold acclimation (4/2°C, 14-h light/10-h dark photoperiod, 250 μmol m^-2^ s^-1^ PAR).

Rubisco activity was higher in treated MFT plants than in the controls after the prehardening and cold acclimation steps (Figure 4). In the LFT plants, 3 weeks of cold acclimation induced an increase in RA over the control levels. RA was unaffected by the treatment in LFT plants after the prehardening step and after 1 week of cold acclimation. The RA of the control LFT plants was unchanged for the duration of the experiment. In contrast, RA in treated LFT plants was stable until the end of the first week of cold acclimation, but increased between the end of the first and third weeks of cold acclimation. In MFT control plants, RA decreased after 2 weeks of the experiment, and increased at the end of the experiment. Meanwhile, the treated MFT plants exhibited a decrease in RA between C0 and the end of the first week of cold acclimation, and an increase in RA between 1 and 3 weeks of cold acclimation.

**FIGURE 5:**
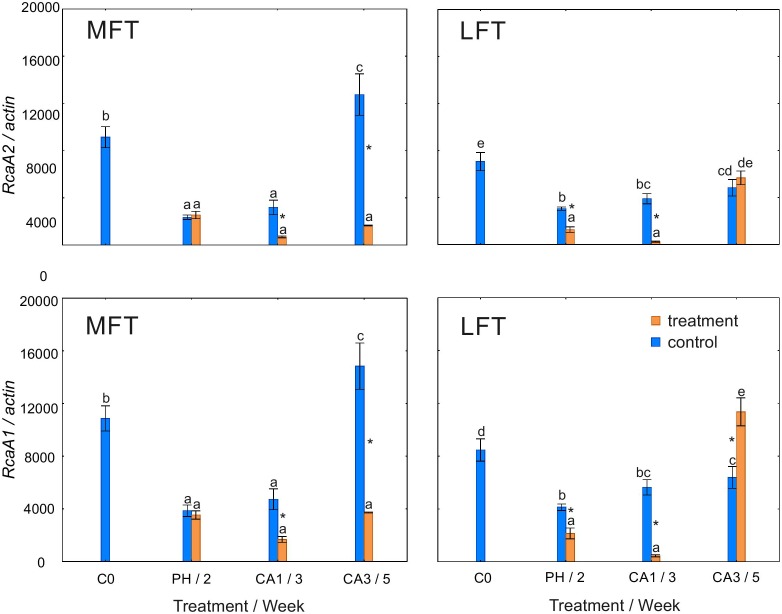
Changes in the relative expression levels of *Rca*A1 and *Rca*A2 encoding Rubisco activase isoforms in *Hordeum* *vulgare* plants after the prehardening and cold acclimation steps. Data are presented as the mean ± standard error. Asterisks indicate values that are different between the treated and control plants according to a one-way ANOVA (*P* = 0.05). Values marked with the same letter are not significantly different (*P* = 0.05) according to Duncan’s multiple range test. LFT and MFT, low and high freezing-tolerant plants, respectively. Measurements were taken before the prehardening step (C0; 18°C, 14-h light/10-h dark photoperiod, 250 μmol m^-2^ s^-1^ PAR), after 2 weeks of prehardening (PH/2; 15/12°C, 14-h light/10-h dark photoperiod, 250 μmol m^-2^ s^-1^ PAR), and after 1 (CA1/ 3) and 3 (CA3/5) weeks of cold acclimation (4/2°C, 14-h light/10-h dark photoperiod, 250 μmol m^-2^ s^-1^ PAR).

In MFT plants, similar changes in *Rca*A1 and *Rca*A2 transcript levels were observed during the experiment (Figure 5). In control plants, the transcript abundance for both gene variants decreased after 2 weeks of the experiment, but increased at the end of the experiment. Plants exposed to cold stress had lower *Rca*A1 and *Rca*A2 transcript levels after the prehardening step than at C0. The transcript levels were then stable until the end of the experiment. Additionally, the transcript abundance for both gene variants was lower in cold-acclimated plants than in the controls. In control LFT plants, *Rca*A1 and *Rca*A2 transcript levels decreased relative to the C0 levels after 2 weeks (Figure 5). Between the second week and the end of the experiment, the transcript levels increased slightly. In LFT plants that underwent a low-temperature treatment, the *Rca*A1 and *Rca*A2 transcript levels decreased between C0 and the end of the prehardening step. In contrast, the transcript levels increased between 1 and 3 weeks of cold acclimation. A decrease in transcript abundance relative to the control levels was detected for both genes after the prehardening step and after the first week of cold acclimation. At the end of the experiment, the *Rca*A1 transcript accumulated more in cold-acclimated plants than in the controls. This difference was not observed for the *Rca*A2 transcript.

### PCA Analysis

**FIGURE 6:**
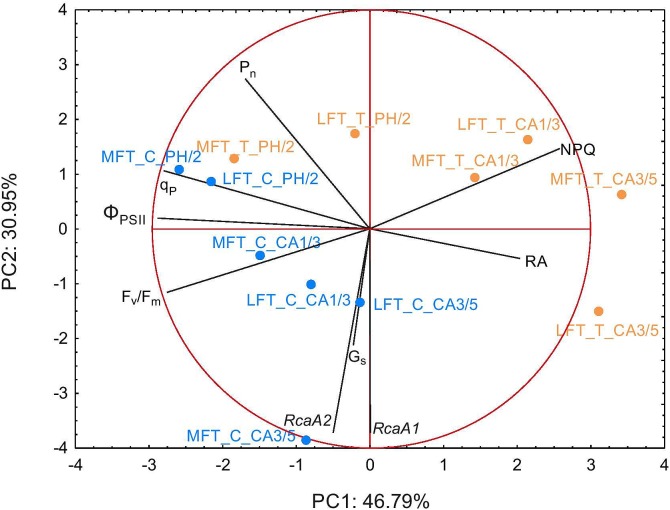
Biplot of principal component analysis (PCA) of physiological traits performed separately after prehardening step (C0; 18°C, 14-h light/10-h dark photoperiod, 250 μmol m^-2^ s^-1^ PAR), after 2 weeks of prehardening (PH/2; 15/12°C, 14-h light/10-h dark photoperiod, 250 μmol m^-2^ s^-1^ PAR), and after 1 (CA1/3) and 3 (CA3/5) weeks of cold acclimation (4/2°C, 14-h light/10-h dark photoperiod, 250 μmol m^-2^ s^-1^ PAR). C and T refers to control and treatment conditions, respectively, LFT and MFT, low and high freezing-tolerant plants.

Principal component analysis used two principal components (PC) and linear combination of variables to take into account a high amount of variance in the data set (77.7% of variation was explained, Figure 6). The structure of observation between different terms of treatment was changed, revealing cold acclimation progress and emphasizing prehardening effect. After prehardening MFT line was related with photosynthetic parameters (P_n_, q_P_, and Φ_PSII_). Such relation also occurred in LFT line, however was not as strong, as for MFT line. During cold acclimation (CA1/3) both lines were transferred toward NPQ vector and against F_v_/F_m_ vector, suggesting activation of non-photochemical mechanism of photosynthetic acclimation to cold. In that time both lines were against G_s_ and *Rca*A vectors. After prolonged cold acclimation (CA3/5) MFT and LFT lines diverged, during that time LFT line was related with RA, but negatively related with other photosynthetic parameters (Figure 6). PCA indicated that G_s_ was positively related with *Rca*A and RA was negatively related with other photosynthetic parameters (P_n_, q_P_, and Φ_PSII_).

## Discussion

Stomatal closure in response to low temperatures limits leaf dehydration in chill-tolerant plants (Wilkinson et al., 2001). The underlying mechanism occurs in cold-tolerant, but not cold-sensitive plants. In this study, G_s_ was lower in cold-acclimated plants than in the controls for both studied barley lines, but differences between the two lines were observed after the prehardening step. Decreases in G_s_ were related to changes in Φ_PSII_. A decrease in CO_2_ input (and concomitant decreases in ATP and NADPH consumption) may have adversely affected the efficiency of electron transport. Decreases in G_s_ and Φ_PSII_ after the prehardening step in treated plants relative to control were detected only in LFT plants. Therefore, moderate cold stress induced stomatal closure (after prehardening) only in plants with a relatively low freezing tolerance. The LFT barley plants likely exhibited stress symptoms (dehydration) at earlier time points. In contrast, in MFT plants, G_s_ did not decrease after the prehardening step, while RA increased. A previous study revealed that prehardening adaptations related to carbon metabolism likely enhance cold acclimation effects (Rapacz and Janowiak, 1998). These adaptations may be dependent on CO_2_ availability.

Principal component analysis revealed that G_s_ and *Rca*A variables are related. A limited CO_2_ concentration may downregulate *Rca*A expression. In MFT plants, a decrease in *Rca*A transcript levels after 1 and 3 weeks of cold acclimation may be a consequence of a relative lack of CO_2_. In LFT plants, this transcript-level effect occurs earlier (i.e., after the prehardening step) and is maintained until 1 week after initiating the cold acclimation. However, after 3 weeks of cold acclimation, the *Rca*A transcript abundance increased, especially *Rca*A1 (encoding the large Rubisco isoform). In the LFT plants, upregulated expression of both analyzed gene variants was observed between the first and third weeks of cold acclimation. The proportion of the transcripts also changed, resulting in a relatively high *Rca*A1/*Rca*A2 transcript ratio. During this time period, RA increased considerably and as indicated by PCA this variable was related with LFT line. Interestingly, P_n_ did not increase, as in MFT plants. These results suggest that the activation of Rubisco helps to protect the photosynthetic machinery by consuming the excess energy rather than by increasing photosynthetic capacity. Additionally, during this time period, a decrease in CO_2_ concentration may favor photorespiration, which may help to protect photosynthetic apparatus from photoinhibition. Consequently, at the end of the experiment, the LFT plants had recovered from photoinhibition.

Freezing tolerance is reportedly related to the tolerance to cold-induced photoinhibition (Rapacz et al., 2004, 2011). In the current study, the MFT plants were more photoinhibited than the LFT plants. Interestingly, after the cold acclimation, photoinhibitory conditions were associated with an increase in P_n_. As previously noted (Hüner et al., 1993), photoinhibition is not a process that inevitably damages photosynthetic apparatus, but is related to the capacity of plants to adjust photosynthetic activities.

Principal component analysis revealed that during cold acclimation treated plants are transferred toward NPQ vector and against G_s_ vector suggesting activation of NPQ mechanism. That activation likely corresponds to stomata decrease. The detailed data showed that the LFT and MFT plants differed in the dynamics of NPQ increases. The LFT plants activated the underlying mechanism earlier (after prehardening) than in the MFT plants [during cold acclimation (i.e., a more severe cold stress)]. Increases in NPQ may be triggered by changes in G_s_. Under insufficient CO_2_ concentrations, the main metabolic sink is limited and the balance in the energy flow of photosynthetic apparatus may be disturbed. This may act as a signal for increasing NPQ to maintain cellular homeostasis. An early NPQ activation may indicate that LFT plants are particularly sensitive to photoinhibition.

A relationship between freezing tolerance and the activation of NPQ was established by Humphreys et al. (2007). They observed that NPQ increased more in freezing-tolerant genotypes. In contrast, we determined that NPQ increased faster in LFT plants than in MFT plants. An important factor inducing NPQ increases is the carbohydrate status. Increases in NPQ are induced in plants that accumulate carbohydrates (fructans) before winter. Therefore, the relationship between freezing tolerance and NPQ increases may be more pronounced in plants that accumulate large amounts of carbohydrates (before winter), such as perennial grasses, but not young barley seedlings.

Both RCA isoforms reportedly activate Rubisco (Shen et al., 1991; Salvucci et al., 2003). Furthermore, the small isoform is more abundant under control (i.e., unstressed) conditions (Rundle and Zielinski, 1991; Jurczyk et al., 2015). However, in a relatively freezing-tolerant *L. perenne* genotype, a prehardening step was observed to upregulate the expression of the gene encoding the large isoform (Jurczyk et al., 2016a), likely to support photochemical acclimation of photosynthetic apparatus to cold conditions. In the current study, we observed that the photochemical mechanism was not induced after a prehardening step, and *Rca*A expression levels were not upregulated, and were even downregulated in LFT plants. Both of these studies suggest the induction of the photochemical mechanism during photosynthetic acclimation to cold stress is related to the expression of *Rca*A variants. After a prehardening step, the relatively freezing-tolerant *L. perenne* genotype exhibits upregulated expression of the transcript representing the large isoform (Jurczyk et al., 2016a). In the treated LFT *Hordeum* *vulgare* plants, the transcript levels for both gene variants decreased relative to control levels, while in MFT plants, the abundance of both transcripts remained at control levels. Therefore, the induced expression of both isoforms during the prehardening step may also be related to the freezing-tolerance level.

Rubisco activity increased throughout the study period in MFT plants, but only after 3 weeks of cold acclimation in the LFT plants. The induced photosynthetic enzyme activities due to cold acclimation have been reported for winter cultivars or relatively hardy genotypes (Hurry et al., 1995; Jurczyk et al., 2016a). In the present study, the increase in RA in MFT plants was related to an increase in P_n_, but was not associated with the activation of the photochemical mechanism underlying photosynthetic acclimation to cold conditions.

**FIGURE 7:**
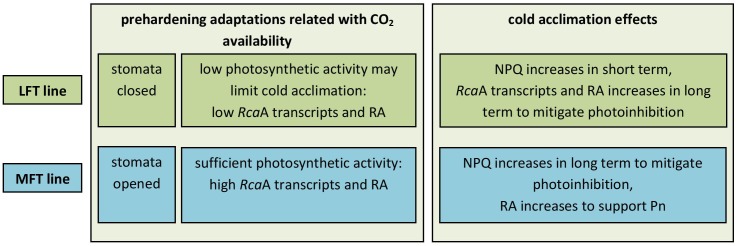
A model of barley response to low temperatures related with CO_2_ availability.

In summary, after a prehardening step, G_s_ decreased in LFT plants, resulting in decreased photochemical activity, downregulated *Rca*A expression and faster NPQ increase. In contrast, the prehardening of MFT plants maintained G_s_ at control levels, leading to delayed photochemical activity and delayed *Rca*A expression decrease, and increased RA supporting P_n_ (Figure 7). In conclusion, the induction of a non-photochemical photoacclimation mechanism (photoinhibition avoidance strategy) is insufficient for an effective cold acclimation, and an increase in P_n_ in response to cold conditions, which is related to the maintenance of open stomata, is necessary.

## Author Contributions

BJ designed the experiments, obtained funding, invited collaborators, measured PSII efficiency, supervised the gene expression studies, analyzed the data, interpreted the results, and wrote the main text of the manuscript. MG measured P_n_ and G_s_ and helped to write a draft of the manuscript. EP measured the RA and helped to write a draft of the manuscript. MW helped to measure *>RcaA1* and *Rca*A2 expression levels. MR was involved in designing the study, interpreting the results, and critically revising the manuscript.

## Conflict of Interest Statement

The authors declare that the research was conducted in the absence of any commercial or financial relationships that could be construed as a potential conflict of interest.

## Abbreviations

F_v_/F_m_: maximum quantum yield of PSII
G_s_: stomatal conductance
LFT: barley line with relatively low freezing tolerance
MFT: barley line with relatively high freezing tolerance
NPQ: non-photochemical quenching coefficient
P_n_: net photosynthetic rate
PSII: photosystem II
q_P_: photochemical quenching coefficient
RA: Rubisco activity
Φ_PSII_: quantum yield of electron transport in PSII
