# Supplementary material for: Diverse Stomatal Behaviors Mediating Photosynthetic Acclimation to Low Temperatures in Hordeum vulgare
Source: Front Plant Sci. 2019 Jan 9;9:1963. doi: 10.3389/fpls.2018.01963 (PMC6333868; doi:10.3389/fpls.2018.01963)
Supplement: Supplementary file 1 [file Table_1.DOCX]

Table 1. Infrared (IRGA) gas analyzer raw data.

| time | treatment | lines | Pn | Gs |
| --- | --- | --- | --- | --- |
| C0 | control | MFT | 9,7 | 34 |
| C0 | control | MFT | 9,9 | 23 |
| C0 | control | MFT | 10 | 36 |
| C0 | control | MFT | 10,3 | 20 |
| C0 | control | MFT | 11 | 27 |
| C0 | control | MFT | 10,3 | 28 |
| C0 | control | MFT | 10,1 | 27 |
| C0 | control | LFT | 10,2 | 28 |
| C0 | control | LFT | 10,9 | 24 |
| C0 | control | LFT | 10,2 | 27 |
| C0 | control | LFT | 11,3 | 29 |
| C0 | control | LFT | 10,5 | 23 |
| C0 | control | LFT | 10,7 | 27 |
| C0 | control | LFT | 10,7 | 29 |
| PH/2 | control | MFT | 9,6 | 65 |
| PH/2 | control | MFT | 8,5 | 51 |
| PH/2 | control | MFT | 9,4 | 53 |
| PH/2 | control | MFT | 9,2 | 58 |
| PH/2 | control | MFT | 8,3 | 58 |
| PH/2 | control | MFT | 8,7 | 76 |
| PH/2 | control | MFT | 9,1 | 72 |
| PH/2 | treatment | MFT | 9,4 | 82 |
| PH/2 | treatment | MFT | 9,3 | 50 |
| PH/2 | treatment | MFT | 9,5 | 63 |
| PH/2 | treatment | MFT | 9,8 | 60 |
| PH/2 | treatment | MFT | 9,8 | 70 |
| PH/2 | treatment | MFT | 8,7 | 63 |
| PH/2 | treatment | MFT | 10,2 | 75 |
| PH/2 | control | LFT | 9,4 | 91 |
| PH/2 | control | LFT | 8,4 | 87 |
| PH/2 | control | LFT | 8,6 | 69 |
| PH/2 | control | LFT | 8,6 | 54 |
| PH/2 | control | LFT | 8,6 | 83 |
| PH/2 | control | LFT | 8,9 | 76 |
| PH/2 | control | LFT | 8,4 | 45 |
| PH/2 | treatment | LFT | 8,1 | 41 |
| PH/2 | treatment | LFT | 8,7 | 61 |
| PH/2 | treatment | LFT | 7,2 | 38 |
| PH/2 | treatment | LFT | 6,7 | 36 |
| PH/2 | treatment | LFT | 9,1 | 57 |
| PH/2 | treatment | LFT | 9,4 | 59 |
| PH/2 | treatment | LFT | 7,9 | 71 |
| CA1/3 | control | MFT | 7,4 | 145 |
| CA1/3 | control | MFT | 7,1 | 170 |
| CA1/3 | control | MFT | 7,1 | 173 |
| CA1/3 | control | MFT | 7,3 | 162 |
| CA1/3 | control | MFT | 8 | 144 |
| CA1/3 | control | MFT | 6,5 | 87 |
| CA1/3 | control | MFT | 6,6 | 68 |
| CA1/3 | treatment | MFT | 6,6 | 100 |
| CA1/3 | treatment | MFT | 6,1 | 60 |
| CA1/3 | treatment | MFT | 6,6 | 154 |
| CA1/3 | treatment | MFT | 7,1 | 51 |
| CA1/3 | treatment | MFT | 6,2 | 50 |
| CA1/3 | treatment | MFT | 6,7 | 76 |
| CA1/3 | treatment | MFT | 6,8 | 67 |
| CA1/3 | control | LFT | 7,7 | 199 |
| CA1/3 | control | LFT | 7,9 | 175 |
| CA1/3 | control | LFT | 6,9 | 184 |
| CA1/3 | control | LFT | 8,4 | 130 |
| CA1/3 | control | LFT | 7,1 | 193 |
| CA1/3 | control | LFT | 6,9 | 114 |
| CA1/3 | control | LFT | 7,4 | 100 |
| CA1/3 | treatment | LFT | 9,3 | 146 |
| CA1/3 | treatment | LFT | 9,4 | 165 |
| CA1/3 | treatment | LFT | 6,7 | 142 |
| CA1/3 | treatment | LFT | 7 | 79 |
| CA1/3 | treatment | LFT | 7,8 | 66 |
| CA1/3 | treatment | LFT | 7,6 | 59 |
| CA1/3 | treatment | LFT | 6,9 | 51 |
| CA3/5 | control | MFT | 5,1 | 105 |
| CA3/5 | control | MFT | 5,5 | 120 |
| CA3/5 | control | MFT | 5 | 94 |
| CA3/5 | control | MFT | 6,8 | 102 |
| CA3/5 | control | MFT | 5,9 | 122 |
| CA3/5 | control | MFT | 6,1 | 111 |
| CA3/5 | control | MFT | 6,2 | 119 |
| CA3/5 | treatment | MFT | 6,8 | 79 |
| CA3/5 | treatment | MFT | 6,6 | 63 |
| CA3/5 | treatment | MFT | 6,9 | 62 |
| CA3/5 | treatment | MFT | 6,6 | 72 |
| CA3/5 | treatment | MFT | 6,5 | 107 |
| CA3/5 | treatment | MFT | 6,3 | 93 |
| CA3/5 | treatment | MFT | 6,7 | 91 |
| CA3/5 | control | LFT | 7 | 191 |
| CA3/5 | control | LFT | 6,8 | 187 |
| CA3/5 | control | LFT | 7,3 | 200 |
| CA3/5 | control | LFT | 6,9 | 186 |
| CA3/5 | control | LFT | 7,7 | 133 |
| CA3/5 | control | LFT | 6,7 | 211 |
| CA3/5 | control | LFT | 7,1 | 199 |
| CA3/5 | treatment | LFT | 6,4 | 67 |
| CA3/5 | treatment | LFT | 7,2 | 68 |
| CA3/5 | treatment | LFT | 7,5 | 55 |
| CA3/5 | treatment | LFT | 6,1 | 67 |
| CA3/5 | treatment | LFT | 6,3 | 86 |
| CA3/5 | treatment | LFT | 6,6 | 87 |
| CA3/5 | treatment | LFT | 7 | 67 |
